# Speed-Dependent Cellular Decision Making in Nonequilibrium Genetic Circuits

**DOI:** 10.1371/journal.pone.0032779

**Published:** 2012-03-13

**Authors:** Nuno R. Nené, Jordi Garca-Ojalvo, Alexey Zaikin

**Affiliations:** 1 Department of Mathematics, Imperial College London, London, United Kingdom; 2 Departament de Fsica i Enginyeria Nuclear, Universitat Politècnica de Catalunya, Terrassa, Spain; 3 Institute for Women's Health and Department of Mathematics, University College London, London, United Kingdom; Queen's University Belfast, United Kingdom

## Abstract

Despite being governed by the principles of nonequilibrium transitions, gene expression dynamics underlying cell fate decision is poorly understood. In particular, the effect of signaling speed on cellular decision making is still unclear. Here we show that the decision between alternative cell fates, in a structurally symmetric circuit, can be biased depending on the speed at which the system is forced to go through the decision point. The circuit consists of two mutually inhibiting and self-activating genes, forced by two external signals with identical stationary values but different transient times. Under these conditions, slow passage through the decision point leads to a consistently biased decision due to the transient signaling asymmetry, whereas fast passage reduces and eventually eliminates the switch imbalance. The effect is robust to noise and shows that dynamic bifurcations, well known in nonequilibrium physics, are important for the control of genetic circuits.

## Introduction

**Figure 1:**
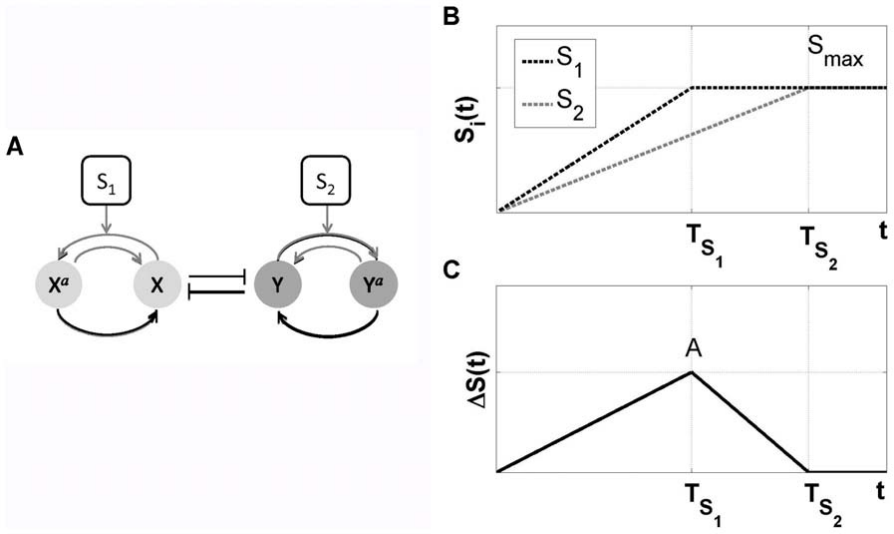
Paradigmatic integrated signaling–transcriptional circuit switch. (A) Schematic representation: Nodes represent proteins, regulated by protein kinases with concentrations 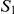 and 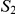, where 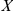 and 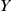 stand for transcription factors that can be phosporylated to generate 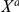 and 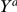. Black lines represent transcriptional interactions, while grey lines stand for protein-protein interactions. (B) Time evolution of the input signals 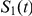 (black) and 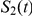 (grey), with 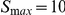. In this work 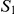 is considered to have a rising time 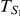 smaller than 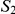. (C) Amplitude of the transient asymmetry between signals 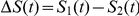. Here the maximal asymmetry is 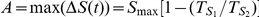 (see also Table 1 and Methods).

Cellular decision making is an inherently nonlinear process requiring multistability, a common feature in nonequilibrium physical systems. This process is driven by gene and protein circuits, which are fundamental for the regulation of many cellular processes, including cell differentiation [1], maintenance of pluripotency [2], developmental pattern formation [3], [4], apoptosis [5], and cell dedifferentiation leading to cancer [6]. In cellular decision making, the cell is forced to decide between alternative fates depending on extracellular conditions. A common circuit that sustains decision making is one in which the two master regulators of the two competing fates inhibit each other, while self-activating themselves in order to increase the stability of the decision outcome (Fig. 1A) [7]. When the interactions are sufficiently symmetric with respect to the two master regulators, this circuit exhibits bistability which is associated with two distinct cell fates and is the focus of our work. In fact, in order for the cell to be able to flexibly choose either of the two fates depending on the conditions, mutually inhibitory cellular decision circuits do need to be as structurally symmetric as possible. Therefore, knowledge of the intracellular circuit structure is insufficient to explain the outcome of phenotype selection. In this situation, external signals may provide the bias necessary for the bistable circuit to fall into one attractor or the other. However, it seems unlikely that the signals will be maintained asymmetric in the long term. Thus the question still remains, as to how does a consistent bias emerge from a symmetric bistable switch subject to signals that are symmetric in the steady state. Here we show that differences in the speed at which the input signals reach their (common) steady state are enough to provide a transient asymmetry that will bias the bistable switch in a consistent manner. In our model, this *speed-dependent cellular decision making* (SdCDM) arises from the inclusion of time-dependent bifurcation parameters, similarly to dynamic bifurcations in applied mathematics [8] and ramped nonequilibrium phase transitions in statistical physics [9]. Since external signals are clearly time-dependent and unlikely to emerge at the same rate in different pathways, we can expect SdCDM to play an important role in many cellular decision-making processes.

## Results

### External signals induce symmetry breaking and transition to bistability

As mentioned above, we study a paradigmatic decision circuit consisting of two mutually repressive proteins under the action of two signals 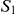 and 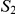 (Fig. 1A), and equate attractor selection with cell fate decision. The proteins, 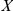 and 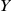, represent transcription factors (TFs) that, when phosphorylated (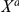, 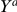) and subsequently dimerized (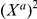, 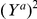), activate their own expression and repress that of the other TF. Many pairs of transcription factors have been shown to act according to this mechanism, including GATA1 and PU.1 in haematopoietic cell differentiation [1], Cdx2 and Oct3/4 in embryonic stem cell differentiation [10], and Pax2 and Pax4 in visual cell specification [11], and it is current practice to construct the models accordingly [7] (see Methods section).

**Figure 2:**
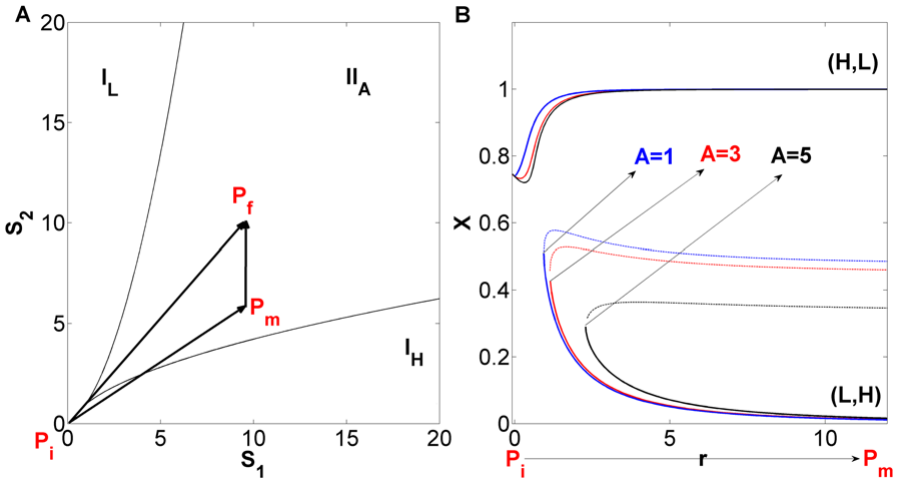
Parameter analysis of the decision switch with external stimulation. (A) Phase diagram for 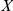 in the space (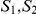). Thin lines represent borders between different regimes: 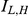 stands for monostability, with 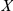 having a low or a high value, respectively. 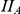 denotes bistability between two states at which 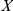 and 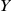 have opposite concentrations, (high, low) or (low, high). 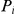, 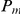 and 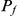 correspond to the initial (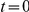), intermediary (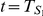), and final (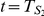) points of the signaling (see Fig. 1B). (B) Bifurcation diagram for protein concentration 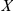 vs parameterization of path 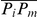, for several values of the maximal asymmetry 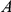 (see Fig. 1C). 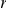 stands for the distance between the origin and a point along the path 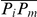. Parameters are 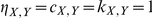 and 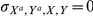 (see also Table 1 and Eq. (1) to (4) in Methods).

With the intent of determining the number of attractors existing for a certain combination of input signals, we performed bifurcation analysis of the circuit using the software XPPAUT [12]. In order to focus on the asymmetry provided by the external signals, the values of all parameters associated with transcription or translation processes are assumed symmetric (see Eqs. (1) to (4) and Table 1 in Methods section). Bifurcation analysis shows that in the parameter space 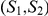 (Fig. 2A) the system can be either monostable (regimes 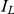 and 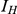) or bistable (regime 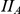). The action of the two external signals takes the system from a state where the cell is undecided (point 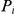) to a situation of bistability (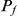), where the system ends up in one of two possible states, which defines the result of cellular decision making. If the two signals 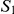 and 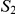 are identical and evolve in time at equal rates, the cell undergoes a transition to bistability through the straight path 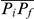. Everywhere along this path there is complete symmetry, and consequently the cell will choose its fate stochastically between the two equally possible stable states.

**Table 1:** Parameters in the decision genetic switch with external stimulation model.

Parameter	Interpretation	Value
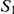	External signal 1	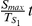 , 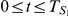 and 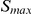 , 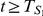
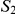	External signal 2	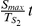 , 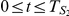 and 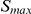 , 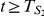
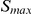	Maximum amplitude of 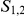	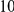
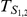	Rising times of 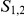	–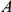	Maximum asymmetry between 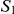 and 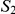	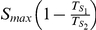
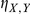	Basal transcription rate multiplied by translation rate divided by 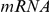 and protein degradation rates	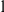
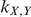	Ratio between binding and unbinding affinities of dimers to promoter regions for self-activation and cross-inhibition, respectively	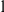
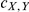	Ratio between rate of expression of the respective gene when homodimers are bound and basal transcription	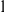
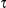	Combined dimensionless time scale for transcription and translation of proteins	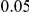 and 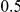
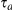	Dimensionless time scale for phosphorylation processes	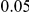
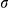	Intensity of Gaussian noise 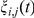 with zero mean and 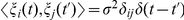	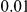 and 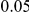

Parameters used in Eqs. (1) to (4) and their respective interpretation and values. See also [7], [15].

The situation changes qualitatively if we consider that the two external signals grow at different rates, which is a more realistic situation. In this case the cellular decision path is 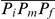, along which the steady states follow an asymmetric bifurcation diagram, as shown in Fig. 1B. At the final decision point (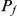, 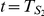) one of the branches (depending on which signal is the fastest, here the top branch) is the preferred one. Although the asymmetry generated when the system follows 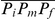 is transient (see Fig. 1C), the memory of the bias induced in the vicinity of the critical region is retained even if the circuit's structure, the initial conditions and the end stationary signaling state are completely symmetric.

### Cell fate decision depends on the speed of passage through the critical region in the presence of fluctuations

**Figure 3:**
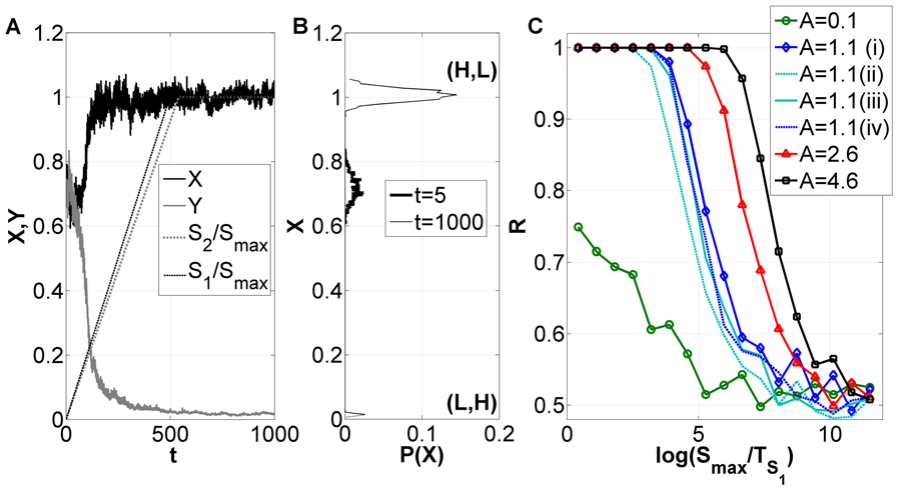
Asymmetric decision under fluctuations. (A) Typical time series of 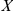 and 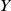 for two input signals that grow at different speeds. (B) Initial and final distribution functions of 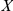 values for 1000 cells. (C) Dependence of the fraction 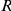 of cells that end up in the high branch, on the speed of the transition (measured by 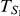) for different values of the maximum asymmetry 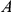 (see Fig. 1C). For all curves in (A), (B) and (C) with exception of plot 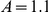 (ii), (iii) and (iv), the underlying equations are Eqs. (1) to (4) with 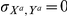. Also shown in (C) for 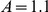 are the ratios 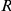 for an extended version of the system of Eqs. (1) to (4) with noisy 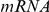 dynamics (dashed dark blue line, no symbols (iv)) and without noise (solid light blue line, no symbols (iii)) (see also Eqs. (6) to (9) in Methods). We also tested the effects of fluctuations in phosphorylation reactions, i.e. 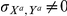 (see (C), dashed light blue line, no symbols 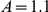 (ii)) for the extended system of equations (Eqs. (6) to (9)) with noisy 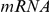 dynamics. Parameters for (A), (B) and (C) 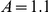 (i) are those of Fig. 1 and 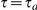 for all curves (see also Table 1 and Methods). Parameters for (C) 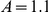 (ii), (iii) and (iv) are 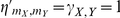 and 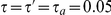 (see also Eqs. (6) to (9)). For all curves 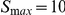 and where fluctuations are considered 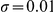.

The behavior described above is robust to noise. Figure 3A shows a typical time series of the circuit, when the signals increase linearly as described in Fig. 1B. We used a Heun method [13], [14] for integrating the differential Eqs. (1) to (4). The distribution of 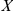 values for 1000 different realizations of the dynamics is shown in Fig. 3B for two time points. Initially the distribution is unimodal and starts to broaden until the saddle-node bifurcation (Fig. 2B) is reached. At that point a bimodal distribution emerges, which is strongly asymmetrical due to the transient signaling asymmetry, with the upper branch being much more populated than the lower one.

We now study the effect of the signaling speed on the decision. To that end, we vary the value of 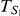 and 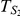 according to 
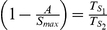
, while keeping the parameters 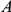 and 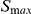 constant (see Fig. 1C and the respective caption and Table 1). In that way, we change the speed of the transition but keep constant the decision path 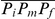 in the parameter space 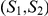. The result is plotted in Fig. 3C, in terms of the fraction 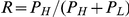 of realizations ending in the upper state (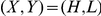, see Fig. 2B). The plot evidences the existence of SdCDM, since for slow signaling speed 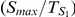 the decision is asymmetric, while the decision becomes unbiased when the signaling speed becomes large enough, with the ratio 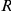 tending to 0.5. It can also be observed that higher values of maximum asymmetry 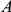 between 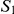 and 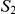 induce a stronger bias in the decision, i.e. higher 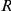 ratios (Fig. 3C).

For comparison purposes we also show in Fig. 3C the ratio 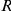 calculated when, in addition to the processes represented in Eqs. (1) to (4), 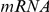 dynamics are considered, both in the absence (solid light blue line, no symbols, 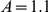 (iii)), and in the presence (dashed dark blue line, no symbols, 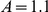 (iv)) of fluctuations (in the latter case, noise is introduced following the same rationale of Eq. (11)). In these simulations, the 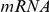 dynamics was assumed to be dependent on transcription initiation (see Eq. (10)) following a function similar to 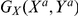 in Eq. (5), and the protein dynamics was considered to depend linearly on 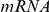 concentration (see Eqs. (6) to (9)). Both species are subject to linear degradation terms [7], [15], and have equal time scales (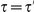). We observe that SdCDM persists in the presence of 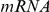 dynamics, although a shift towards smaller 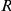 ratios appears when compared with the original case in which only protein dynamics was considered (Fig. 3C, solid dark blue line, diamond symbols 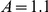 (i)). Overall, the extra steps degrade, but only slightly, the probability of reaching the upper branch due to a delay emerging from the 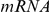 dynamics. We also tested for the extended system (Eqs. (1) and (2) and (6) to (9)) the effects of fluctuations in the phosphorylation reactions (Fig. 3C, dashed light blue line 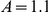 (ii)). Again, during the simulations the noise term was calculated through an expression that follows the rationale of Eq. (1). The extrinsic noise source present when fluctuations are included in phosphorylation reactions degrades additionally the ratios 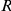, but still elicits SdCDM. Further investigations are necessary to clarify the effect of extrinsic noise [16] on SdCDM and to establish the limits of sensitivity of SdCDM to intermediate steps (see for example [17], [18]).

**Figure 4:**
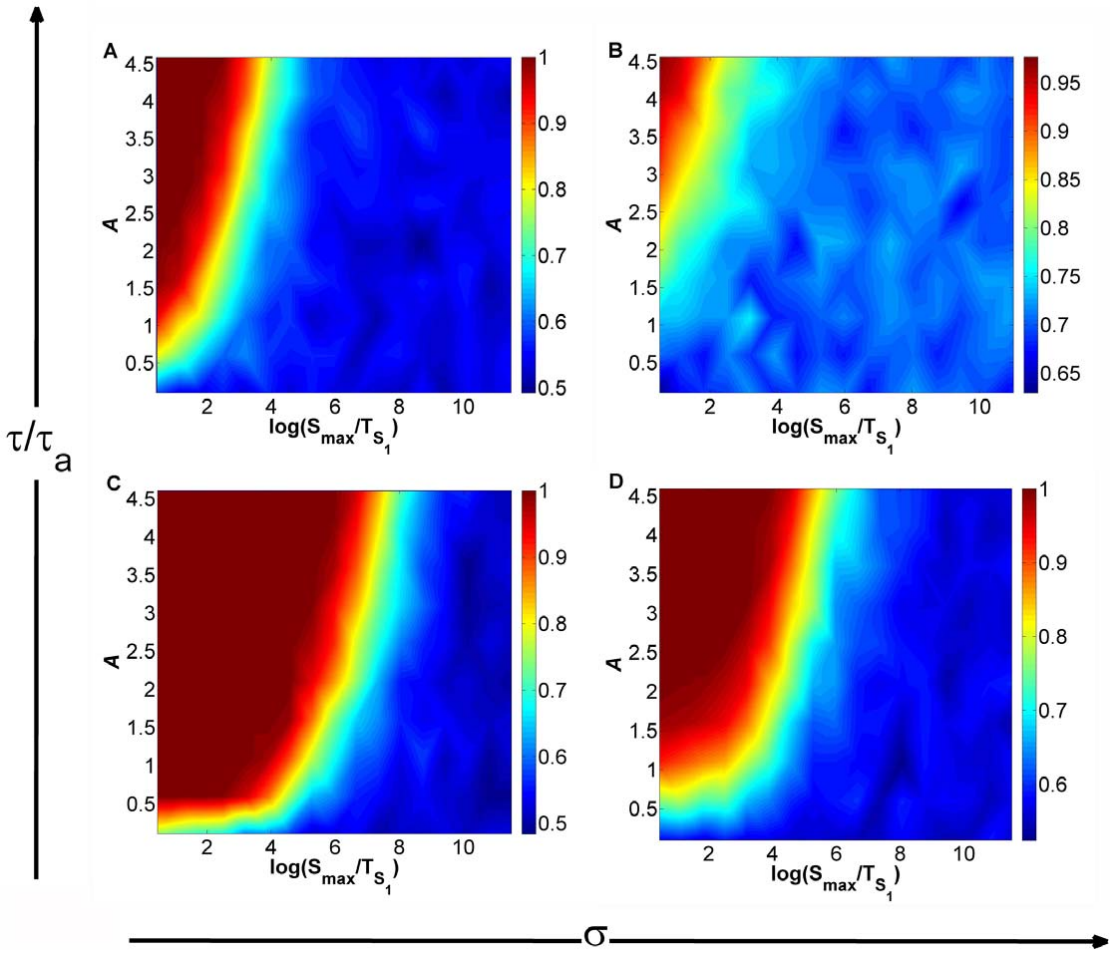
Effect of time scale differences and noise on SdCDM. The decision bias, measured by the fraction 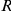 of cells that end up in the high branch, is plotted in color scale versus the transient asymmetry parameter 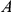 and the signaling speed (controlled by 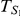), for several noise and time scale ratios. (A) 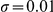 and 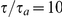. (B) 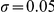 and 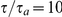. (C) 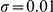 and 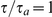. (D) 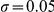 and 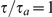. Blue denotes a symmetric decision, and red an asymmetric decision, which appears for slow enough speeds if the transient asymmetry is non-zero. Parameters are those of Fig. 1, plus 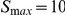 (see also Table 1 and Methods). The underlying equations are Eqs. (1) to (4) with 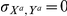.

So far we have considered that the time scales of phosphorylation and production of transcription factors were equal. Yet, extracellular signals usually change the activity state of transcription factors in a sub-second scale, while transcription and translation of target genes may take minutes, and accumulation of protein products minutes to hours, with the additional delay being due to macromolecular transport [19]. To understand the effect of different time scale ratios 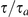 and noise on the decision bias, we performed extensive numerical simulations of Eqs. (1) to (4) on a 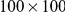 grid of combinations of maximum asymmetry 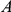 and signaling speed 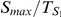, with 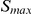 at a constant value (see Methods section and Table 1). For simplicity we focused only on the case where no fluctuations in phosphorylation reactions are present, and the 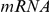 dynamics were assumed again to be in a quasi-steady state. The results are shown in Fig. 4, and clearly reveal that as the difference in the phosphorylation and expression/translation time scales increases, and also as the noise intensity 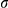 grows, the ability of the decision circuit to choose consistently a cell fate depending on the signaling speed decays.

## Discussion

The mechanism of SdCDM can be understood from previous studies in dynamic bifurcations and parameter sweeping experiments in physical systems [8], [9]. As in the case of the generic bistable potential [9], [20], the speed at which the system crosses the critical region strongly influences the sensitivity to the transient asymmetry (see Fig. 4). Although the signaling is symmetric in the steady state, during the signal build-up the circuit is momentarily exposed to asymmetric signals. With high speed the system is able to ignore this transient asymmetry, but slow enough sweeping speeds increase the probability of reaching one of the steady states over the other one, because they induce a smaller bifurcation delay [21]–[23]. Bifurcation delays arise when the system takes a long time to leave the neighborhood of the unstable state. In the case of large bifurcation delays the probability that the system jumps across the potential barrier is increased, and consequently the circuit capacity to discriminate signals 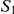 and 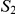 is reduced. On the other hand, higher maximum asymmetries between 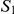 and 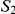 reduce the bifurcation delays and also the amplification of fluctuations [9], [22]. Similarly to [9], the probability of biasing the distribution depends on the maximum amplitude of the asymmetry to noise ratio, on the one side, and inversely on the sweeping speed and time scale ratio, on the other. This can be seen when comparing for instance Figs. 4A and 4C; it is clear that the loss of bias in the final distributions caused by an increase in the time scale ratio can be compensated by decreasing also the sweeping speed controlled by 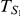. Graphically, this means that cross sections of 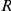 versus 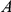 (for constant 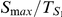) in Fig. 4A are similar to those observed in Fig. 4C when shifted to the left by approximately 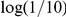.

Certain cell differentiation processes are driven by slow build-up of decision-driving signals [24]. A mechanism of temporal control of differentiation has also been proposed [25], and experiments have revealed that temporal competition can determine cell fate choice in multipotent differentiation [26]. Here we have shown that the speed of signaling in genetic switches dramatically changes the result of cellular decision, an effect that we have termed speed-dependent cellular decision making (SdCDM). In contrast to other aspects of nonequilibrium physics [27]–[29], dynamic bifurcations have not been systematically studied in systems biology despite involving fundamental aspects of cell fate decision. It is of special interest in this context because all genetic switches are asymmetric and stochastic and, hence, can be expected to demonstrate SdCDM. Our study extends the well-known delayed bifurcation effect in physics to a wide class of equations used to model gene expression. This will be of importance for understanding the dynamics of genome-wide networks and meets the recent interest and relevance of delayed dynamics in fields such as developmental biology [30]. In contrast to previous studies, in our work asymmetry in signaling/genetic network models is *transient* and *non-additive*. It is an open question which additional dynamics can appear due to the interplay between speed of asymmetry emergence and speed of decision making. It would also be interesting to understand, through analytical techniques, the importance of reaching the maximum asymmetry 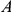 (see Fig. 1C) before or after the bifurcation point and establish a parallel with the canonical bistable potential [20].

We can conjecture that evolutionary adaptation has provided embryonic development with the optimal speed for cellular differentiation and, consequently, deviation from this speed may lead to pathologies. The conditions leading to such anomalies, and their potential treatment, constitute still an important open question. The mechanism demonstrated here should have further impact in investigations of genetic circuits with high dimension and undergoing more complex types of bifurcation [31]. Also, since both subcritical and supercritical pitchfork bifurcations can explain decision making in cell differentiation (see e.g. lineage-commitment in bipotent blood progenitor cells [1]), it should be interesting to determine how the type of the bifurcation will affect SdCDM. Experimental differentiation studies, with special emphasis on pattern formation, constitute also a viable avenue that is expected to reveal interesting relationships between the speed at which the system grows, and the organized complexity permitted in morphogenesis [3].

## Methods

The dynamics of the protein concentrations involved in our circuit (see Fig. 1A) is described by a phenomenological model following [7], [15] and assumed to be dimensionless:
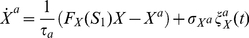
(1)

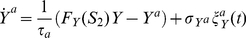
(2)

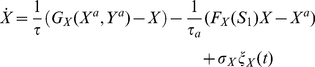
(3)

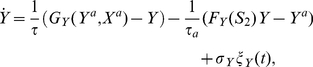
(4)In this model, Eqs. (1) and (2) represent the phosphorylation-dephosphorylation dynamics, where the latter is assumed to occur with a constant rate (corresponding to a constant phosphatase concentration, a common assumption in pathway modeling [32]). Phosphorylation, on the other hand, is considered to depend on the external signals. Following Fig. 1A this process is represented by: 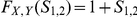, with the unit term standing for basal activation. The transcriptional input of 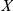 contains the stimulatory action of its phosphorylated form 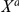 and the inhibitory effect of 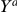, and is modeled according to a mean-field approach to promoter site occupation [15] (see Eq. (5)).
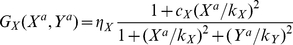
(5)


Respectively for the protein 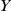. Both TFs are assumed to act as homodimers, a usual situation in real systems [33]. The parameters 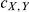 represent the ratio between the maximally activated expression rate and basal transcription, while 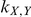 denote ratios associated with activation and repression thresholds. The parameters 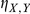 are a measure of the promoter strength multiplied by translational efficiency [15]. Finally, the characteristic time scales of phosphorylation and protein expression are given by 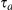 and 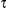, respectively (see also Table 1).

Eqs. (1) to (4) were derived by assuming that transcription factor binding and unbinding, on the one hand, and 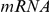 dynamics, on the other, are fast when compared to protein dynamics [7], [15], [19]. Although there is also a substantial difference between the time scales of translation and phosphorylation events [19], the profile of activation of each transcription factor or of signals 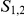 has been proven to be fundamental to understand cell fate decision [34]–[36]. Therefore, we maintained the activation Eqs. (1) and (2). Moreover, this option allows us to extend in further studies the impact on cell fate decision, here equated with attractor selection, of partial inhibition of phosphorylation processes exerted by specific classes of drugs [37]. Most of the results on SdCDM presented in this work follow the system of Eqs. (1) to (4) with 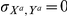. Yet, for comparison purposes we extended part of the results of Fig. 2C (curve 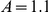 (i)) to include 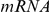 dynamics (curves 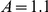 (ii), (iii) and (iv), see Results section), where Eqs. (3) and (4) are substituted by Eqs. (6) to (9), with or without noise in 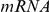 and phosphorylation dynamics.
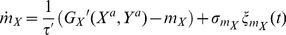
(6)

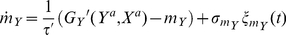
(7)

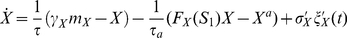
(8)

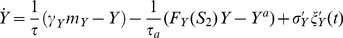
(9)


In Eqs. (6) and (7) the functions 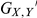 follow a similar expression to Eq. (5), with adjusted parameters 
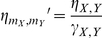
 (see Eq. (10) and also Table 1 for comparison with the reduced model), but continue to set the model as dimensionless. Parameters 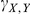 (see Eq. (8) and (9)) represent translation.
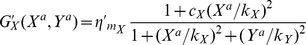
(10)


Correspondingly for 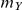. Both the reduced model (Eqs. (1) to (4)) and the extended one (Eqs. (6) to (9)) assume that the circuit operates in a constant-volume cell. In the case of the reduced model we take into account stochastic fluctuations only in gene expression [38], i.e. 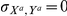 (see Fig. 3 A, B, 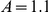 (i) plot in C and Fig. 4). To that end, 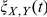 represents a Gaussian noise with zero mean and correlation 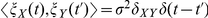, and models the contribution of intrinsic random fluctuations inherent to transcription and translation processes [16]. The multiplicative noise term in Eqs. (3) and (4) is interpreted according to Ito, which is the correct stochastic interpretation for a noise term arising from stochastic protein-gene interaction events [39]. Accordingly, the noise intensity functions 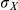 and 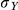 that appear in Eqs. (3)–(4) take the following form [39] (see Eq. (11)).
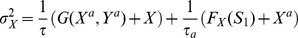
(11)


Correspondingly for protein 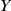. For the parameters 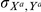 in Eq. (1) and Eq. (2) and 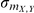 and 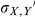 in Eq. (6) to Eq. (9), wherever it was computed (see Fig. 3C, plots 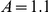 (ii), (iii), (iv)), the procedure that led to Eq. (11) was once more applied [39] and 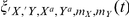 also represent Gaussian noise with intensity 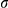.

All bifurcation diagrams (see Fig. 1) were created in XPPAUT [12]. Parameter 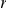 in Fig. 1B can be determined by 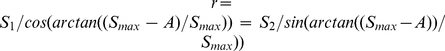, where 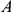 stands for the maximum asymmetry reached between 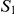 and 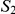 (see Fig. 1C).

Furthermore, all simulation results were performed by numerically integrating the stochastic differential equations using the Heun method [14] with a scaled time-step of 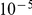. Each simulation was started at the steady state available for 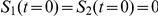 and subsequently the external signals 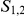 were changed linearly until reaching the maximum value allowed (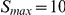, see Fig. 1B). In order to calculate the ratio 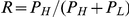 (see Fig. 3), the set of simulations was performed until an instant 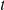 far beyond 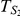 to secure that the system had converged.

With respect to Figs. 4A to D, the results were generated by sampling a 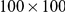 matrix of the 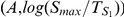 space and fitting a surface, through the 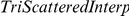 linear interpolation method (MATLAB R2010b), to the numerical data obtained.
